# Prevalence of histopathological subtypes associated with steroid-resistant nephrotic syndrome in children: a systematic review and meta-analysis

**DOI:** 10.3389/fimmu.2025.1647608

**Published:** 2025-11-20

**Authors:** Sen Lin, Shanshan Zheng, Wanying Dong, Yangyang Tian, Hao Li, Xinyuan Gao, Fang Qin, Chaoqun Ma, Yipeng Liu

**Affiliations:** 1 Department of Nephrology, The First Affiliated Hospital of Shandong First Medical University & Shandong Provincial Qianfoshan Hospital, Shandong Institute of Nephrology, Jinan, China; 2 Department of Nephrology, Shandong Provincial Qianfoshan Hospital, Shandong University, Jinan, China; 3 Department of Nephrology, Shandong Second Provincial General Hospital, Jinan, China; 4 Department of Emergency Medicine, Shandong Provincial Hospital Affiliated to Shandong First Medical University, Jinan, China

**Keywords:** steroid-resistant nephrotic syndrome in children, histopathologic subtypes, focal segmental glomerulosclerosis (FSGS), prevalence, immune-mediated

## Abstract

**Background:**

Steroid-resistant nephrotic syndrome (SRNS) in children is associated with various histopathological subtypes;however, the prevalence of these subtypes remains insufficiently defined. These subtypes often demonstrate variable responses to identical therapeutic regimens and are indicative of distinct clinical outcomes. This systematic review and meta-analysis seeks to comprehensively elucidate the prevalence of the most common histopathological subtypes in pediatric SRNS, offering valuable insights that may inform future therapeutic strategies and improve prognostic predictions for affected patients.

**Methods:**

We conducted a comprehensive literature search across four major databases: PubMed, Embase, Web of Science, and Cochrane, with coverage extending from the inception of these databases up to December 2023. Two independent researchers screened the identified studies, selecting only cross-sectional and longitudinal studies that reported the prevalence of histopathological subtypes in pediatric SRNS. The quality of the studies was assessed using the Joanna Briggs Institute Critical Appraisal Checklist, and only those meeting the required quality criteria were included. Data were extracted from the selected studies using a standardized data extraction form. Meta-analysis was performed with Stata software to estimate the prevalence of different histopathological subtypes in pediatric SRNS. Subgroup analyses were conducted to explore potential sources of heterogeneity. Publication bias was assessed using funnel plots and the Begg test, and sensitivity analysis was also conducted.

**Results:**

The most commonly reported histopathological subtypes associated with SRNS in children are focal segmental glomerulosclerosis (FSGS), membranous nephropathy (MN), minimal change disease (MCD), mesangioproliferative glomerulonephritis (MesPGN), and membranoproliferative glomerulonephritis (MPGN). Among these, the subtype with the highest average prevalence is FSGS, at 39%, followed by MCD (27%), MesPGN (23%), MPGN (18%), and MN (4%).

**Conclusion:**

FSGS exhibits a notably high prevalence in SRNS and remains the most frequently observed histopathological lesion associated with this condition.

**Systematic review registration:**

https://www.crd.york.ac.uk/PROSPERO/view/CRD420251000869, identifier CRD420251000869.

## 1 Introduction

The prevalence of common histopathological subtypes associated with steroid-resistant nephrotic syndrome (SRNS) in children appears to be undergoing global changes, and different histopathological subtypes often exhibit divergent responses to the same therapeutic regimens (1). Nephrotic syndrome (NS) is characterized by massive proteinuria, hypoalbuminemia, hyperlipidemia, and edema. Many pediatric kidney diseases manifest as nephrotic syndrome, and for the majority of patients who respond to steroid treatment, the primary therapeutic approach involves the use of prednisone. However, a certain proportion of patients fail to respond to steroid treatment, a phenomenon known as steroid resistance. And frequent relapses may occur during treatment, necessitating the use of alternative immunosuppressive agents. Steroid resistance is a critical factor in the progression of pediatric nephrotic syndrome to chronic kidney disease or end-stage renal disease. NS can be classified based on response to steroid therapy, relapse patterns, histopathological findings, or genetic mutations. The simplest classification is based on clinical response to steroid treatment, distinguishing between steroid-sensitive (SS) and steroid-resistant (SR) forms. SRNS is defined as the failure to achieve complete remission after four weeks of treatment with prednisone at standard doses (60 mg/m²/day or 2 mg/kg/day) in nephrotic syndrome patients.

SRNS is a clinically diagnosed disease characterized by histological features identified through kidney biopsy. The treatment of SRNS remains a significant challenge for nephrologists. Although renal biopsy is often indicated in children with SRNS, few large-scale studies have evaluated the role of histopathology in the contemporary era (2), where genetic diagnoses are feasible (3), or in the presence of obstacles such as contraindications to biopsy, lack of nephropathology expertise, or unavailability of electron microscopy. Minimal change nephrotic syndrome (MCNS) typically shows no significant abnormalities on light microscopy, whereas electron microscopy reveals diffuse foot process effacement of podocytes. This subtype is most commonly observed in children aged 2–5 years. Approximately 80-90% of MCNS cases respond to steroid treatment and are classified as steroid-sensitive nephrotic syndrome (SSNS).In contrast, SRNS patients often exhibit FSGS on kidney biopsy. Light microscopy reveals segmental obstruction of the glomerular capillaries due to extracellular matrix expansion, causing encapsulation and sclerosis of plasma proteins. The electron microscopy findings in FSGS typically resemble those of MCNS, with diffuse effacement of podocyte foot processes.

Steroids are the most commonly used treatment for nephrotic syndrome in clinical practice. However, different histopathological subtypes often predict varying outcomes and responses to steroid therapy. Some patients with idiopathic nephrotic syndrome may undergo a series of potentially harmful treatments without clinical benefit (1). Previous study have indicated a high prevalence of focal segmental glomerulosclerosis(FSGS) in children with nephrotic syndrome in sub-Saharan Africa (4), making it the most common histopathological subtype of SRNS worldwide. However, due to the limited number of studies included in that review, a meta-analysis has not yet been performed. To the best of our knowledge, no meta-analysis has been published on the prevalence of pathological subtypes in children with SRNS. In this study, we conducted a systematic literature search to extract data on the prevalence of histopathological subtypes and performed a meta-analysis to determine the prevalence of each subtype. Our findings will provide valuable insights for guiding clinical treatment decisions and predicting outcomes in children with SRNS.

## 2 Methods

A comprehensive literature search was conducted in PubMed, Embase, Web of Science, and Cochrane databases from their inception to December 2023, adhering to the PRISMA (Preferred Reporting Items for Systematic Reviews and Meta-Analyses) flowchart to identify relevant studies for this systematic review. The search was systematically performed using Boolean operators (OR and AND) to combine key terms. The search strategy was based on three primary concepts: “steroid,” “nephrotic syndrome,” and “drug resistance.”

This review was conducted in accordance with the guidelines of the PRISMA statement for systematic reviews and meta-analyses. Additionally, this study has been registered with PROSPERO, registration number: CRD420251000869.

### 2.1 Search strategy

#### 2.1.1 Databases searched

We conducted a comprehensive literature search across PubMed, Embase, Web of Science, and the Cochrane Library, covering the period from database inception through December 2023, to identify studies relevant to the objectives of this systematic review. All retrieved records were manually screened for eligibility.

#### 2.1.2 Search terms and search strategy

A systematic search was conducted using Boolean operators (OR and AND) to combine key terms. The search strategy was based on three primary concepts: “steroid,” “nephrotic syndrome,” and “drug resistance.” The search strategy was developed according to the PICOs principle:(1)P (Population): Children with SRNS.(2)I (Intervention) and C (Comparison): Not applicable to this study.(3)O (Outcome): Prevalence of histopathological subtypes in SRNS children or studies reporting the number of SRNS histopathological subtypes to facilitate prevalence calculation.(4)S (Study design): This review includes cross-sectional and longitudinal studies. Non-SRNS populations, qualitative studies, and secondary analyses were excluded. For studies involving both adults and children, only data specific to pediatric SRNS histopathological subtypes were retained. Among multiple articles using the same dataset, the study with the most comprehensive data or the largest sample size was selected.

#### 2.1.3 Inclusion and exclusion criteria

##### 2.1.3.1 Inclusion criteria

**①**SRNS: Defined as the failure to achieve complete remission after 4 weeks of prednisone treatment at standard doses (60 mg/m²/day or 2 mg/kg/day) (5).**②**Kidney Biopsy Results: Studies must report the kidney biopsy findings of the patients.**③**Age Range: Only studies involving children aged 3 months to 18 years will be considered (6).

##### 2.1.3.2 Exclusion criteria

①Case reports, letters to the editor, and review articles were excluded.**②**Studies that did not report kidney biopsy results.**③**Studies involving children with steroid-dependent nephrotic syndrome (SDNS) or frequently relapsing nephrotic syndrome (FRNS) were excluded.

### 2.2 Study screening

The search results were imported into EndNote X7, and duplicates were removed. Two researchers(Sen lin and Wanying Dong) independently screened the titles and abstracts of the identified studies using EndNote X7. At least one researcher reviewed the full text of studies that were potentially eligible for inclusion. The eligibility of each study was assessed by both researchers, and in cases of disagreement, a third researcher(Yangyang Tian) was consulted. Additionally, the reference lists of included studies were manually screened to identify any additional relevant studies that met the inclusion criteria.

### 2.3 Data extraction

Two researchers (Sen lin and Wanying Dong)independently extracted data using a pre-tested data extraction form. Any discrepancies in data extraction were resolved through discussion or by consulting the original publications. The information collected included the title, first author, publication year, study period, country of study, study design, sample size, participant age range, mean age, histopathological subtypes, and their prevalence. Countries were categorized according to the World Bank (WB) classification as high-income, low- and middle-income countries.

### 2.4 Quality assessment

The quality of the included studies was assessed using the Joanna Briggs Institute Critical Appraisal Checklist. Studies meeting the quality criteria were included. Two researchers(Sen lin and Wanying Dong)independently reviewed all included studies, and a third researcher(Yangyang Tian) was involved when necessary to reach a consensus.

### 2.5 Meta-analysis

Data analysis was performed using Stata versions 14.0, 15.0, and 17.0, along with the *meta* and *metafor* software packages. The overall prevalence of histopathological subtypes associated with pediatric SRNS was calculated. Subgroup analysis was conducted based on the income level of the country where the study was conducted. Due to high heterogeneity, a random-effects model (DerSimonian-Laird method) was applied in the meta-analysis to determine the overall prevalence of histopathological subtypes related to pediatric SRNS. Forest plots were generated to visually summarize the details of individual studies and estimate the common effect size, as well as the degree of heterogeneity. Cochran’s Q test was used to assess heterogeneity, and the I² statistic was employe for quantification. I² values of 25%, 50%, and 75% were considered indicative of low, moderate, and high heterogeneity, respectively. Funnel plots were used for a qualitative visual assessment of publication bias, and the Begg rank correlation test was employed for quantitative assessment. Sensitivity analysis was conducted for each histopathological subtype to evaluate the influence of individual studies and assess whether any study had an inordinate effect on the overall results.

## 3 Results

**Figure 1:**
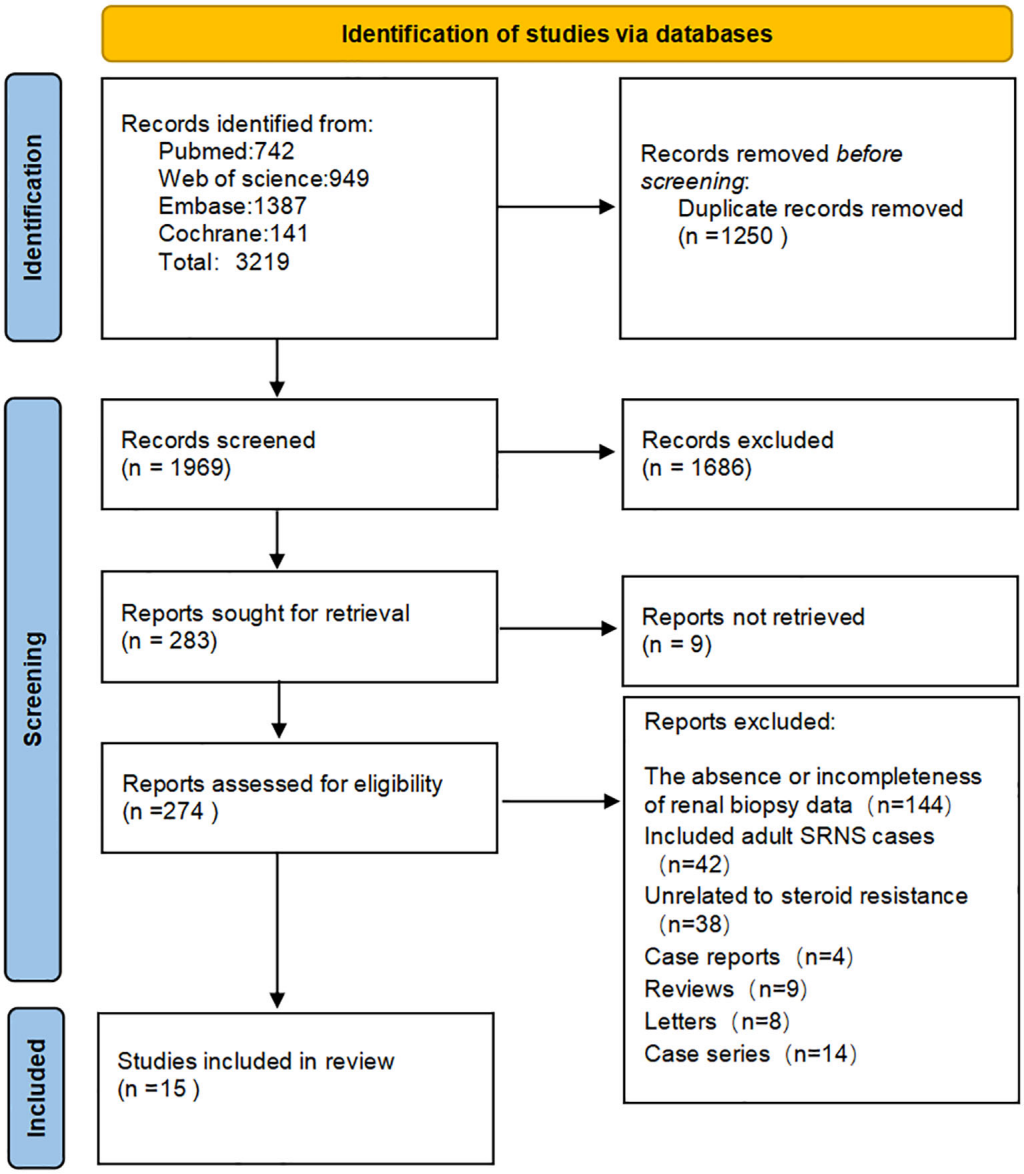
Preferred reporting items for systematic review and meta‐analysis protocols flow diagram. EMBASE, Excerpta Medica database; Cochrane, Cochrane Central Register of Controlled Trials.

The articles included in this review comprised prospective studies, retrospective studies, and randomized controlled trials, focusing on children and adolescents aged 3 months to 18 years. A total of 3,219 articles were initially retrieved, with 1,250 duplicates removed. After reviewing the titles and abstracts of the remaining 1,969 articles, 283 were selected for full-text review. Of these 283 articles, 9 did not have the original text available, and 274 articles were successfully retrieved in full. Upon full-text review, 247 articles were excluded, including those the absence or incompleteness of renal biopsy data(144 articles), case reports (4 articles), reviews (9 articles), studies involving adult SRNS (42 articles), studies unrelated to steroid resistance (38 articles), letters (8 articles), case series (14 articles). Ultimately, 15 studies were included. **Figure 1** shows the PRISMA flowchart of the selection process.

### 3.1 Description of included studies

**Figure 2:**
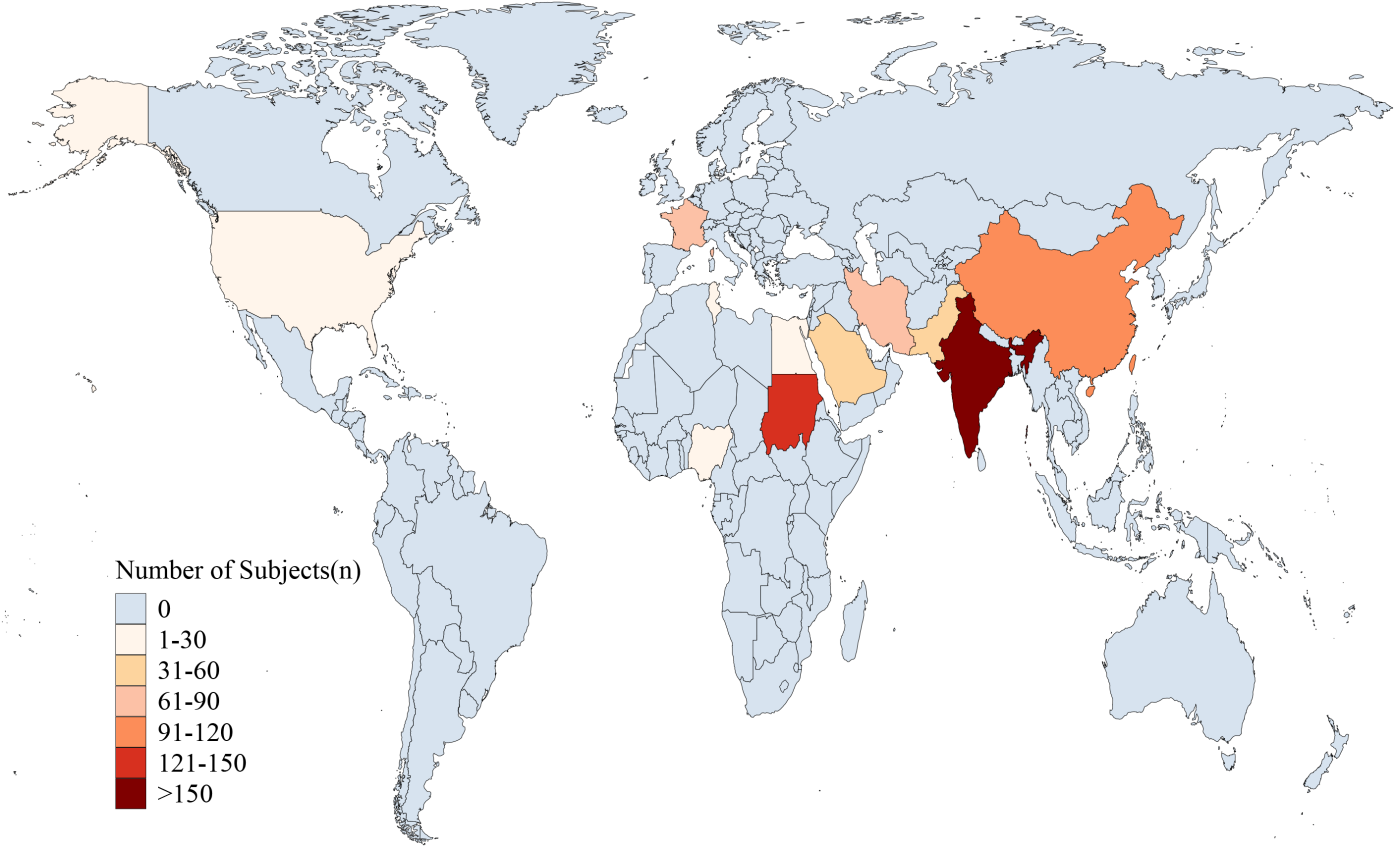
Geographical distribution of study participants. (The participant distribution across the included countries was: Iran (66), India (250), China (100), Kingdom of Saudi Arabia (37), Pakistan (46), Tunisia (30), Nigeria (23), Sudan (130), United States (29), France (78), and Egypt (16).

**Table 1** summarizes the characteristics of the studies included in this review. The studies were conducted in 11 different countries or regions, distributed across Asia [9 studies (7–15)], Africa [4 studies (16–19)] and Europe/North America [2 studies (20, 21)]. The participant distribution across the included countries was: Iran (66), India (250), China (100), Kingdom of Saudi Arabia (37), Pakistan (46), Tunisia (30), Nigeria (23), Sudan (130), United States (29), France (78), and Egypt (16)(**Table 1**, **Figure 2**). The majority of studies were observational (n = 12), while the remaining three studies (7–9) were randomized controlled trials (RCTs). All studies focused on children aged 3 months to 18 years diagnosed with SRNS. Each study reported the prevalence of various histopathological subtypes of SRNS, with five subtypes included in the analysis: FSGS, membranous nephropathy (MN), minimal change disease (MCD), mesangioproliferative glomerulonephritis (MesPGN), and membranoproliferative glomerulonephritis (MPGN). The most commonly reported subtypes were FSGS (n = 15), MCD (n = 14), and MesPGN (n = 14). Eleven studies (7–10, 14–20) were published after 2010. All studies reported prevalence data for both boys and girls. The quality score for each included study was at least 6 points, with the detailed quality assessment provided in the **Supplementary Table 1**. When patient ages followed a normal distribution, they were presented as mean ± standard deviation; otherwise, the median and interquartile range were reported.

**Table 1:** Characteristics of included studies.

No.	Study	Period of study	Sample size	FSGS	MCD	MPGN	MesPGN	MN	Regions	Age,yr(control vs treatment)	Male, n (%)(control vs treatment)	Study design
1	Farahnak Assadi,2022 (7)	2019-2021	66	47	15	None	4	None	Iran	4.2(2.4;5.9) vs 4.3(2.7;5.8)	15(47%) vs 14(41%)	RCT
2	K. M. Shah,2017 (8)	2008-2011	50	13	29	None	8	None	India	4.54±3.46 vs 5.34±2.96	None	RCT
3	Pankaj Hari,2018 (9)	2011-2015	30	9	9	11	None	1	India	12 (8.5, 15.3) vs 12 (10, 14)	11 (73.3) vs 10 (66.7)	RCT
4	Zhihui Li,2010 (10)	2005-2008	24	3	None	2	15	None	China	1.51±0.36	6(25)	Prospective
5	Sanjeev Gulati,2006 (11)	1993-2005	136	80	24	2	24	6	India	8.7±5.1	100(73.5)	Retrospective
6	Tahar Garga,2011 (16)	2002-2009	30	15	9	None	6	None	Tunisia	7.16±4.36	11(36.7)	Retrospective
7	Nammalwar B.R.,2006 (12)	1998-2002	34	10	12	None	12	None	India	None	13 (38.2)	Prospective
8	Wasiu A. Olowum,2010 (17)	2001-2007	23	9	1	10	2	1	Nigeria	8.3 ± 3.5	16(69.6)	Retrospective
9	El-Tigani M. A. Ali,2017 (18)	2001-2012	130	53	21	22	29	5	Sudan	7.7 ± 4.12	78(60)	Retrospective
10	Jameela A. Kari,2009 (13)	2002-2007	37	14	3	None	6	None	Saudi Arabia	4.3 ± 3.0	25(69.4)	Retrospective
11	Caroline Straatmann,2013 (20)	2002-2009	29	7	19	None	2	None	USA	None	19(65.5)	Retrospective
12	Djalila Mekahli,2009 (21)	1988-2008	78	33	35	None	10	None	France	4.4(1.1;15.0)	45(58)	Retrospective
13	Yasser Gamal,2016 (19)	2014-2015	16	9	5	None	2	None	Egypt	13.1 ± 1.5	9(56.3)	Prospective
14	Hai-Xia Chen,2019 (14)	2015-2017	76	17	1	None	52	1	China	7 ± 3	53(69.7)	Prospective
15	Khemchand Netaram Moorani,2019 (15)	2005-2017	46	21	12	7	None	6	Pakistan	None	None	Retrospective

FSGS, focal segmental glomerulosclerosis; MN, membranous nephropathy; MCD, minimal change disease; MesPGN, mesangioproliferative glomerulonephritis; RCT, randomized controlled trial.

### 3.2 Quality assessment

**Supplementary Table 1** presents the quality assessment results for the included studies. The review performed well on questions 1, 4, 5, 6, 7, and 8, which address the appropriateness of the sample frame, detailed description of study subjects and setting, thorough data analysis, accurate diagnosis, standardization of measurement methods, and proper statistical analysis. However, the review performed poorly on questions 2, 3, and 9, which address the appropriateness of the sampling method, sample size adequacy, and follow-up rate.

### 3.3 Prevalence of histopathological subtypes in SRNS

**Figure 3:**
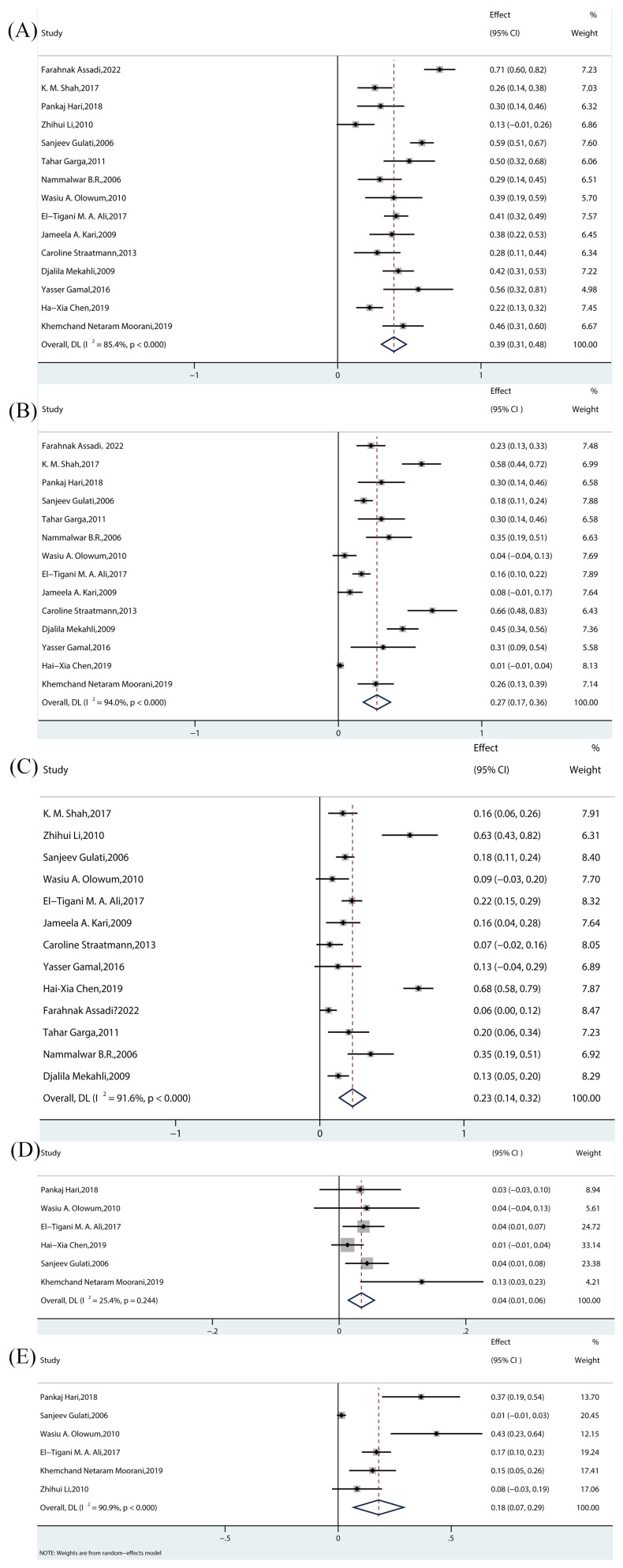
Meta-analyses of the prevalence rates across pathological subtypes. **(A)** Prevalence of FSGS in children. **(B)** Prevalence of MCD in children. **(C)** Prevalence of MesPGN in children. **(D)** Prevalence of MN in children. **(E)** Prevalence of MPGN in children. FSGS, focal segmental glomerulosclerosis; MCD, minimal Change Disease; MesPGN, mesangial proliferative glomerulonephritis; MN, membranous nephropathy;MPGN, membrano-proliferative glomerulonephritis.

The estimated prevalence of histopathological subtypes associated with SRNS is as follows: FSGS: 39% (95% CI, 31-48; I² = 85.4%; p < 0.000) (**Figure 3A**); MN: 4% (95% CI, 1-6; I² = 25.4%; p = 0.244) (**Figure 3D**); MCD: 27% (95% CI, 17-36; I² = 94.0%; p < 0.000) (**Figure 3B**); MesPGN: 23% (95% CI, 14-32; I² = 91.6%; p < 0.000) (**Figure 3C**); MPGN: 18% (95% CI, 7-29; I² = 90.9%; p < 0.000) (**Figure 3E**). The I² statistic reveals high heterogeneity for most SRNS histopathological subtypes, except for MN, which exhibited low heterogeneity (I² < 50%).

#### 3.3.1 FSGS

FSGS was the most commonly reported histopathological subtype, with all 15 studies reporting its prevalence. Among these, 3 experimental studies (7–9)(all randomized controlled trials [RCTs]) reported prevalence rates ranging from 26% to 71.21%. The remaining 12 observational studies (10–21)(including prospective, retrospective, and cross-sectional studies) reported prevalence rates ranging from 12.50% to 58.8%. The pooled effect size was statistically significant [Rate = 39%, 95% CI (31–48), p = 0.00].

#### 3.3.2 MCD

MCD was the second most frequently reported histopathological subtype, with 14 studies including it. Among these, 3 experimental studies (7–9)(all RCTs) reported prevalence rates between 22.7% and 58.00%, while 11 observational studies (11–21)(prospective, retrospective, and cross-sectional) reported prevalence rates ranging from 1.32% to 65.5%. The pooled effect size was statistically significant [Rate = 27%, 95% CI (17–36), p = 0.00].

#### 3.3.3 MesPGN

A total of 13 studies reported the prevalence of MesPGN. Of these, 2 experimental studies (7, 8) (RCTs) reported prevalence rates between 6.1% and 16%, while 11 observational studies (10–14, 16–21) (prospective, retrospective, and cross-sectional) reported rates ranging from 8.7% to 68.42%. The pooled effect size was statistically significant [Rate = 23%, 95% CI (14–32), p = 0.00].

#### 3.3.4 MN

Six studies reported the prevalence of MN. Among these, 1 experimental study (9) (RCT) reported a prevalence rate of 9.68%, and 5 observational studies (11, 14, 15, 17, 18) (prospective, retrospective, and cross-sectional) reported rates between 1.3% and 13%. The pooled effect size was statistically significant [Rate = 4%, 95% CI (1–6), p = 0.244].

#### 3.3.5 MPGN

Six studies reported the prevalence of MPGN. Of these, 1 experimental study (9) (RCT) reported a prevalence rate of 36.6%, while 5 observational studies (10, 11, 15, 17, 18) (prospective, retrospective, and cross-sectional) reported rates between 1.5% and 43.48%. The pooled effect size was statistically significant [Rate = 18%, 95% CI (7-29), p = 0.00].

### 3.4 Subgroup analysis of SRNS histopathological subtypes

From the forest plots of each histopathological subtype, it is evident that most of the subtypes exhibit high heterogeneity. Therefore, we performed subgroup meta-analyses to identify potential sources of heterogeneity. Typically, each subgroup should contain at least three studies. However, it was not possible to group studies by the world health organization(WHO) regional standards, as only two studies (20, 21) belonged to the Europe/North America region, and subgroup analysis could not be conducted for this group. Subgroup analyses by study type showed that heterogeneity remained substantial both before and after subgrouping.

**Figure 4:**
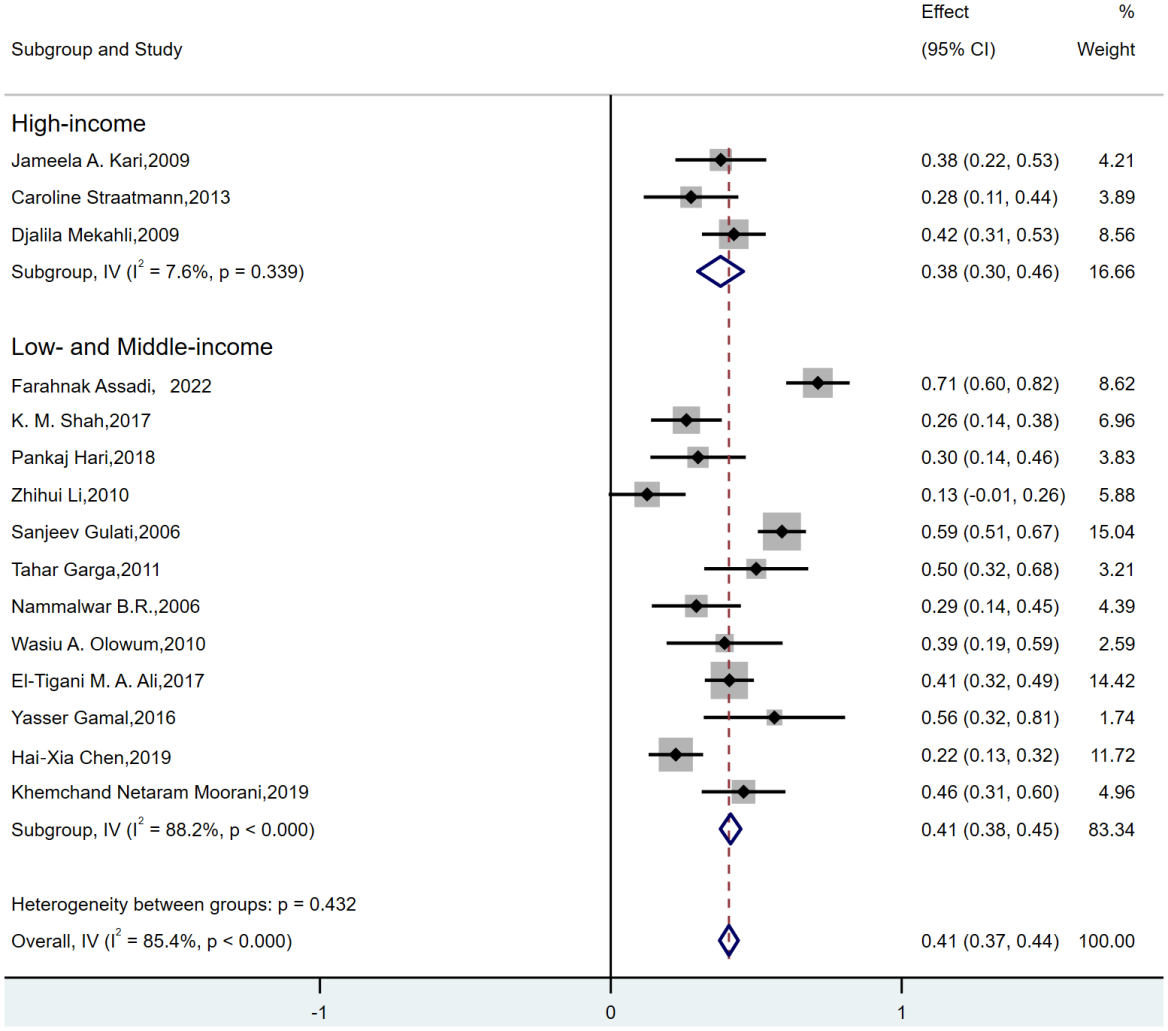
Subgroup analysis of prevalence of FSGS in children by “country income level”.

For FSGS, MCD, and MesPGN, subgroup analyses were conducted based on the income level of the country where the study was conducted (high-income, low-income, and middle-income countries). Since MN exhibited low heterogeneity, no subgroup analysis was conducted for this subtype. Additionally, no studies from high-income countries were available for MPGN, so subgroup analysis was not performed for these two histopathological subtypes. The results showed that when grouping by the income level of the country where the study was conducted, the following observations were made:for FSGS(**Figure 4**), the “low- and middle-income” group (7–12, 14–19) exhibited high heterogeneity (I² = 88.2%, p < 0.000), while the “high-income” group (13, 20, 21) showed low heterogeneity (I² = 7.6%, p = 0.339). Therefore, the heterogeneity in FSGS could be attributed to the “low- and middle-income” populations. For MesPGN (**Supplementary Figure 10**), the “low- and middle-income” group (7, 8, 10–12, 14, 16–19) showed high heterogeneity (I² = 93.2%, p < 0.000), while the “high-income” group showed very low heterogeneity (I² = 0.0%, p = 0.431). This suggests that the heterogeneity in MesPGN might also stem from the “low- and middle-income” populations. For MCD(**Supplementary Figure 11**), heterogeneity remained high both before and after subgrouping based on “country income level”.

### 3.5 Sensitivity analysis

As shown in **Supplementary Figures 5**-**9**, removing any single study for each histopathological subtype did not significantly affect the final pooled effect size, indicating that the results of this study are relatively stable and the findings are highly credible.

### 3.6 Publication bias

**Figure 5:**
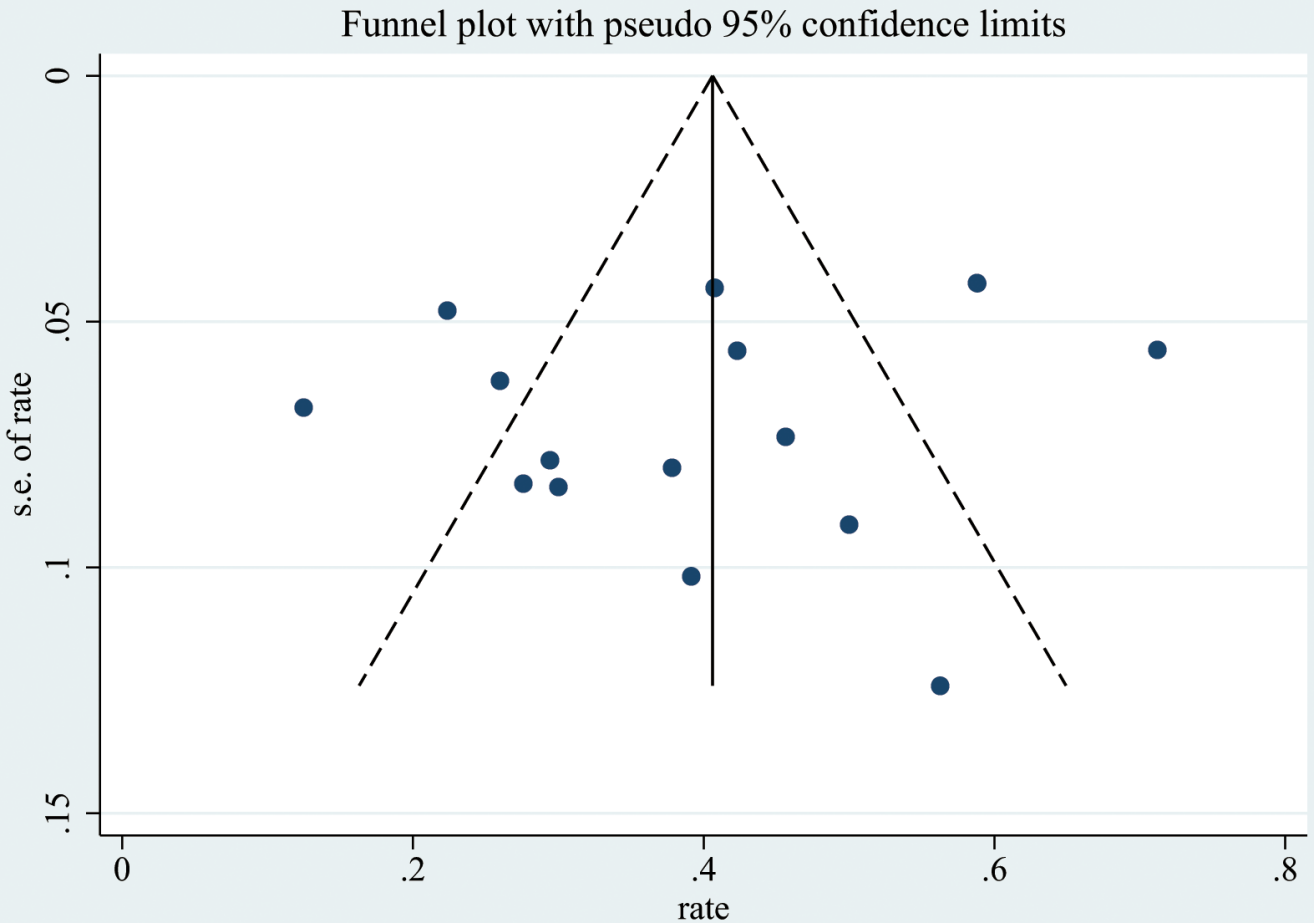
Funnel plot showing no publication bias for the studies reporting FSGS.

Publication bias was qualitatively assessed using funnel plots(**Figure 5**, **Supplementary Figures 1**-**4**). However, assessing the symmetry of funnel plots can be highly subjective. Therefore, we also used the Begg rank correlation test (**Table 2**) for a quantitative assessment. The results indicated that the p-values for the Begg test for FSGS, MesPGN, MN, MCD, and MPGN were all greater than 0.05, suggesting that there is minimal publication bias, and the results are therefore reliable.

**Table 2:** Prevalence rates of histopathologic subtypes SRNS Using Random-Effects Meta-analysis and Subgroup Meta-analysis.

Variable	No. of articles	No. of participants	No. of cases	Prevalence(95% Cl)	I², %	P value		
						Q test	Begg test	Subgroup difference
Global analysis for steroid resistance in childhood nephrotic syndrome								
FSGS	15	805	338	39%,(31-48)	85.4	<0.000	0.843	N/A
MCD	14	781	195	27%, (17-36)	94	<0.000	0.071	0
MesPGN	13	681	171	23%, (14-32)	91.6	<0.000	0.125	0
MN	6	441	20	4%, (1-6)	25.4	0.244	0.26	N/A
MPGN	6	389	55	18%, (7-29)	90.9	<0.000	0.452	N/A

FSGS, focal segmental glomerulosclerosis; MN, membranous nephropathy; MCD, minimal change disease; MesPGN, mesangioproliferative glomerulonephritis; RCT, randomized controlled trial.

## 4 Discussion

Historically, pediatric NS was considered a benign condition with a favorable response to steroid treatment. Morphologically, most cases presented as minimal change glomerulonephritis, which typically had a good long-term prognosis. Over 85% of children and adolescents with NS experience complete remission of proteinuria after steroid therapy (2, 22). A positive response to steroids has long been considered an important prognostic indicator for preserving kidney function. Although many patients with SSNS often experience relapses or develop steroid resistance, their long-term renal prognosis is generally favorable. The main challenge for these patients is the side effects of prolonged corticosteroid therapy and the use of other immunosuppressive agents.

However, for those who do not respond to steroids, a diagnosis of SRNS is made. SRNS is typically diagnosed when there is no complete remission after 4 weeks of prednisone treatment at standard doses (60 mg/m²/day or 2 mg/kg/day). SRNS is considered a significant risk factor for progression to end-stage renal disease (ESRD). Steroid resistance most commonly occurs during the initial period of prednisone treatment (initial resistance), but it may also develop during relapse treatment in patients who previously responded to steroids or second-line therapies (late resistance).

Renal biopsy remains a critical tool for both diagnosis and prognostic classification in nephrology (23). It allows for the assessment of tissue characteristics through optical, immunofluorescent, and electron microscopy techniques. An adequate biopsy typically includes approximately 25 glomeruli, especially when evaluating focal or segmental lesions (22). The pioneering International Study of Kidney Disease in Children (ISKDC), conducted between 1967 and 1976 with patients recruited across three continents, first reported that MCNS was the most common histological finding in kidney biopsies from children with idiopathic NS (24, 25). In contrast, FSGS is a rare cause of childhood NS, with a biopsy positivity rate of only 5%-7% (24, 25). Subsequent studies (22) have reported that in patients with SRNS, the prevalence of FSGS, MCD and MesPGN ranges from 35-55%, 25-40%, and 10-15%, respectively, which aligns closely with the conclusions of our meta-analysis. Our meta-analysis included 15 studies, all of which incorporated FSGS. Seven of these studies consistently identified FSGS as the most prevalent histological subtype associated with SRNS in children. In pediatric nephrology, steroid resistance is often considered a hallmark of FSGS, as clinicians typically resort to renal biopsy to clarify the etiology after corticosteroid treatment failure. Moreover, compared to other histological subtypes, the global prevalence of FSGS appears to be on the rise (43% vs 62% vs 86%; P = 0.03) (26) particularly in Western countries and Asia (27–32). However, these reports have not systematically assessed patients’ steroid responsiveness; therefore, it remains unclear whether the histological changes are concomitant with sustained steroid resistance and long-term prognosis variations. A study conducted in Nigeria indicates that the most common cause of nephrotic syndrome has shifted from quinine-induced nephropathy (QMN) in the 1960s, to MPGN in the 1980s, and now to FSGS (33). A recent review highlighted this trend, suggesting that Nigeria may be undergoing a transition from QMN to MPGN, and now to FSGS (34). The reasons behind the increasing prevalence of FSGS remain unclear. It may be attributed to advancements in diagnostic methods or could reflect an absolute increase in incidence, potentially driven by obesity and chronic inflammation. For instance, the improvement in renal biopsy expertise may contribute to the rising prevalence, as FSGS might be easily overlooked if only cortical glomeruli are biopsied, without sampling medullary glomeruli to capture a more comprehensive picture.

Recently, FSGS has been classified into six forms (35) primary or idiopathic FSGS, adaptive FSGS (the two most common forms), high-mutation genetic FSGS, virus-mediated FSGS, drug-induced FSGS (three less common forms), and the newly identified APOL1-related FSGS. Emerging evidence indicates that FSGS associated with the APOL1 gene is more likely to progress to ESKD. A morphologic classification (36) was previously proposed based on the Columbia classification, which defines 5 variants: collapsing, tip, cellular, perihilar, and not otherwise specified(NOS).FSGS (NOS) represents the most prevalent “classic” variant (36), which is a diagnosis of exclusion established after ruling out other specific subtypes. Its prognosis correlates primarily with the severity of proteinuria and serum creatinine levels at clinical presentation, with nephrotic-range proteinuria portending a poorer outcome. In pediatric populations, this variant is frequently accompanied by diffuse mesangial hypercellularity; however, this finding is generally regarded as an early disease manifestation and does not appear to influence long-term prognosis. The perihilar variant is defined by the presence of perihilar sclerosis and hyalinosis involving greater than 50% of segmentally sclerotic glomeruli. The perihilar variant is strongly associated with secondary forms of FSGS (37), such as those related to obesity or renal hypoplasia, and clinical management should prioritize addressing the underlying etiology. The cellular variant is characterized by segmental endocapillary hypercellularity and typically presents with severe nephrotic syndrome of acute onset. This variant is thought to represent an early, active stage of FSGS, and due to its dynamic histologic features, may demonstrate a more favorable response to immunosuppressive therapy, including corticosteroids, compared to other subtypes. The tip variant of FSGS is defined by the presence of at least one glomerulus with a segmental lesion involving the tip domain (i.e., the peripheral 25% of the glomerular tuft next to the origin of the proximal tubule).The relationship of tip lesion to minimal change disease and FSGS has been hotly debated. Regarding the tip variant, a substantial proportion of studies indicate that affected patients exhibit higher rates of response to corticosteroid treatment and maintain good long-term renal survival, resembling outcomes seen in minimal change disease. Nonetheless, some cases may still progress to classic FSGS. The designation of collapsing variant (also known as collapsing glomerulopathy) is applied to cases of FSGS in which at least one glomerulus displays segmental or global obliteration of the glomerular capillary lumina by wrinkling and collapse of glomerular basement membranes(GBMs) associated with podocyte hypertrophy and hyperplasia. When compared with patients with FSGS (NOS), patients with idiopathic collapsing FSGS are more likely to be black (38) and to present with more severe markers of nephrotic syndrome, including more severe proteinuria, hypoalbuminemia, and hypercholesterolemia. The collapsing variant is typically resistant to corticosteroid therapy. Although the included studies lacked further pathological subtyping of FSGS, we hypothesize, based on existing literature and clinical observations, that the higher prevalence of the collapsing variant may explain the greater propensity for steroid resistance in children with FSGS. This insight offers a promising avenue for future mechanistic investigations. In addition, a considerable proportion of steroid-resistant patients present with MCD, with a prevalence of 27% in our study cohort, second only to FSGS. This finding aligns with previous reports suggesting that histological minimal changes do not always correlate with steroid responsiveness (39) and sometimes progress to FSGS in follow-up biopsies (40, 41).Some studies propose that MCD and idiopathic FSGS are two manifestations of the same disease progression. FSGS is also a consistent feature of massive podocyte loss in animal models and is a common trait of nearly all progressive glomerular diseases. In clinical trials, idiopathic FSGS should be regarded as an advanced stage of disease progression, with a reduced likelihood of response to treatment when compared to the earlier stages of the disease, typically characterized by MCD (42).A study (43) involving repeated biopsies of 171 patients revealed that in 47 cases, 26 (55%) had their diagnosis changed from minimal change disease to FSGS, while 16 of 33 (48%) had their diagnosis changed from mesangioproliferative glomerulonephritis to FSGS.

Through subgroup analysis, we observed no significant difference in the prevalence of FSGS between high-income and middle-low-income countries. However, the prevalence of MCD was notably higher in high-income countries compared to middle-low-income countries, while the prevalence of MesPGN was significantly lower in high-income countries. It has been previously suggested that race or ethnicity may serve as risk factors for chronic kidney disease (CKD) (e.g., higher risks in African Americans, Indigenous populations in Australia, Canada, and the United States, South Asians, and East Asians). However, these associations may be influenced by socioeconomic factors (44, 45). Some studies have also indicated a link between socioeconomic status and systemic diseases that may involve the kidneys, such as systemic lupus erythematosus (SLE) (46). It is essential to clarify the relationship between socioeconomic status and glomerular diseases for various reasons. For instance, economically disadvantaged populations are more vulnerable to other chronic conditions, which may complicate the management of glomerular diseases (47). Low socioeconomic status can also undermine access to routine care, quality of life, and medication adherence, leading to a vicious cycle (48). Studies (49) have confirmed a negative correlation between socioeconomic status and FSGS. Combined with our subgroup analysis results, this may explain the heterogeneity in FSGS pathology subtypes, which may be attributed to the “national income level.”

The prevalence of other pathological subtypes, including MesPGN, MN, and MPGN, was 23%, 4%, and 18%, respectively. Due to the limited number of studies on C1q nephropathy and IgA nephropathy (both with only two studies), a meta-analysis was not conducted for these conditions, and therefore, these two studies were excluded.

This study, through meta-analysis, provides a comprehensive distribution of pathological subtypes in pediatric SRNS, offering valuable insights for clinical diagnosis and treatment. We have elucidated the distribution of different pathological subtypes in children with SRNS. On one hand, this serves as a valuable addition to epidemiological research on SRNS, allowing for informed assumptions regarding the pathological patterns in these patients, which may enhance nephrologists’ understanding of the disease. On the other hand, these findings, combined with guideline recommendations, can support the empirical selection of appropriate therapies, thereby helping to prevent delays in treatment and reduce the risk of adverse drug reactions. However, it is important to note that our study focused on children with SRNS who have already been diagnosed and undergone renal biopsy, mainly examining the distribution of their pathological subtypes. In clinical practice, there are still cases where a renal biopsy is performed without prior treatment. For these patients diagnosed with a particular pathological subtype of nephrotic syndrome, the likelihood of steroid resistance remains uncertain. Therefore, more reliable evidence from clinical studies is needed to guide the diagnosis and treatment of these patients.

## 5 Strengths and limitations

Previous reviews have primarily focused on the distribution of pathological subtypes in pediatric SRNS within specific regions (e.g., sub-Saharan Africa). To the best of our knowledge, this represents the first meta-analysis to elucidate the distribution of pathological subtypes in pediatric patients with SRNS. However, there are several limitations in this study. Although our search encompassed studies on children with SRNS worldwide, the final inclusion of only 15 studies—spanning 3 continents and 11 different countries (**Figure 2**)—was hampered by obstacles such as limited healthcare resources, lack of renal pathology data, and generally low quality of the available literature. Given the current body of literature, relevant data from many countries or regions remain difficult to obtain or are incomplete. Consequently, the geographical coverage of this meta-analysis is limited and insufficient to reflect the global prevalence of pathological subtypes among children with SRNS. Thus, the findings of this study should be regarded as a snapshot of the currently available evidence. Future epidemiological research should prioritize investigating “dark sites”—regions from which no data on this issue are currently available. Among the 15 studies included, only three were RCTs, with the remainder being observational studies. This may introduce potential biases due to non-random sampling methods and insufficient sample sizes in some of the studies. Another limitation is the lack of an exploration into the distribution of pathological subtypes according to gender and age groups. Most of the included studies did not correlate pathological types with age and gender, thus preventing subgroup analyses based on these variables. Furthermore, a notable heterogeneity was observed in the random-effects models for most pathological types in the meta-analysis. Except for the MN studies, which exhibited low heterogeneity, the I² values for most SRNS pathological subtypes were greater than 75%. This level of heterogeneity is commonly observed in meta-analyses of single-arm studies, such as those on disease prevalence by pathological type.

## 6 Conclusion

The most commonly reported pathological subtypes associated with SRNS in children include FSGS, MN, MCD, MesPGN, and MPGN. Among these, FSGS had the highest prevalence, with an average rate of 39%, followed by MCD (27%), MesPGN (23%), MPGN (18%), and MN (4%). This systematic review and meta-analysis is the first to provide a comprehensive description of the global prevalence of common pathological subtypes associated with steroid resistance in pediatric nephrotic syndrome. The findings of this study will serve as a valuable reference for guiding future pharmacological interventions and predicting outcomes in children with SRNS.

## Data Availability

The original contributions presented in the study are included in the article/**Supplementary Material**. Further inquiries can be directed to the corresponding authors.
