# Supplementary material for: Prevalence of histopathological subtypes associated with steroid-resistant nephrotic syndrome in children: a systematic review and meta-analysis
Source: Front Immunol. 2025 Nov 20;16:1647608. doi: 10.3389/fimmu.2025.1647608 (PMC12675457; doi:10.3389/fimmu.2025.1647608)
Supplement: Supplementary file 1 [file DataSheet1.pdf]

Supplementary Figure 1. Funnel plot showing no publication bias for the studies reporting MCD. 2
Supplementary Figure 2. Funnel plot showing no publication bias for the studies reporting MesPGN. 3
Supplementary Figure 3. Funnel plot showing no publication bias for the studies reporting MN. 4
Supplementary Figure 4. Funnel plot showing no publication bias for the studies reporting MPGN. 5
Supplementary Figure 5. Sensitivity analysis in the meta-analysis of FSGS prevalence rates 6
Supplementary Figure 6. Sensitivity analysis in the meta-analysis of MCD prevalence rates 7
Supplementary Figure 7. Sensitivity analysis in the meta-analysis of MesPGN prevalence rates 8
Supplementary Figure 8. Sensitivity analysis in the meta-analysis of MN prevalence rates 9
Supplementary Figure 9. Sensitivity analysis in the meta-analysis of MPGN prevalence rates 10
Supplementary Figure 10. Subgroup analysis of prevalence of MesPGN in children by "country income level." 11
Supplementary Figure 11. Subgroup analysis of prevalence of MCD in children by "country income level." 12

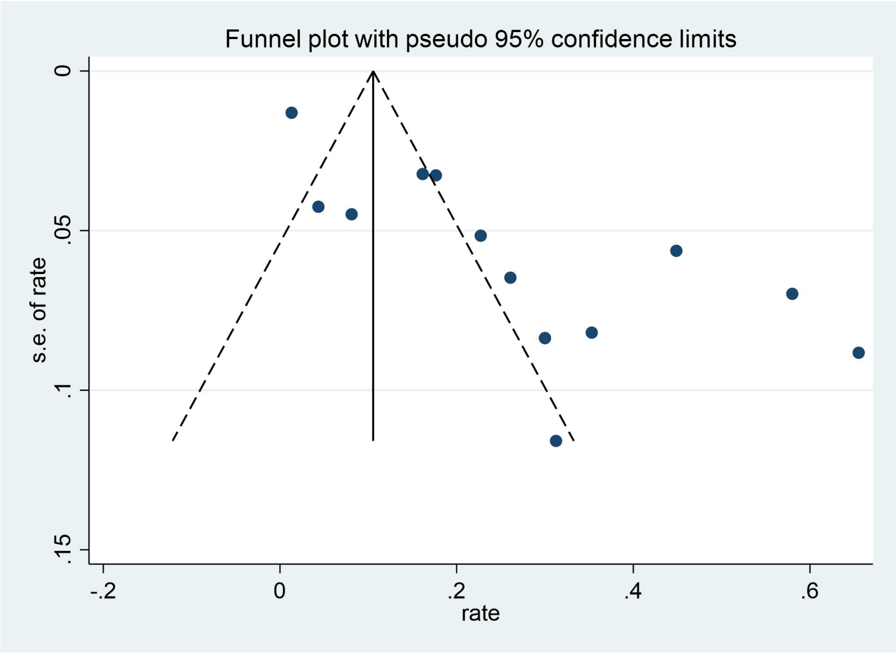

Supplementary Figure 1. Funnel plot showing no publication bias for the studies reporting MCD.

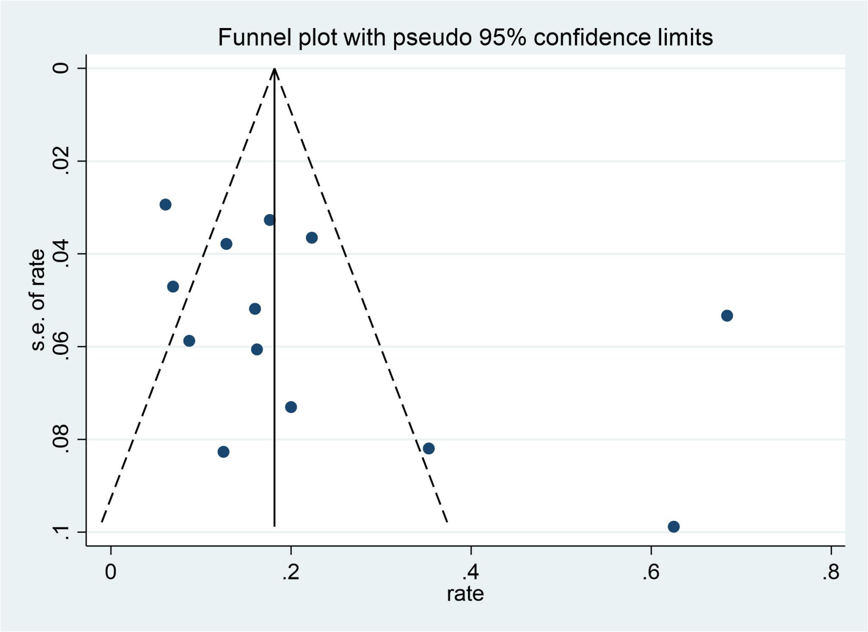

Supplementary Figure 2. Funnel plot showing no publication bias for the studies reporting MesPGN.

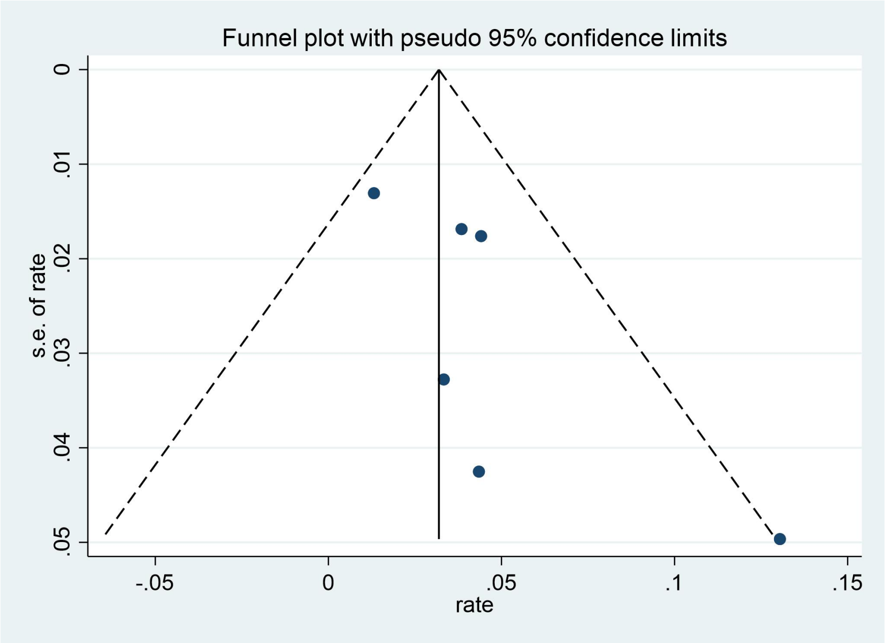

Supplementary Figure 3. Funnel plot showing no publication bias for the studies reporting MN.

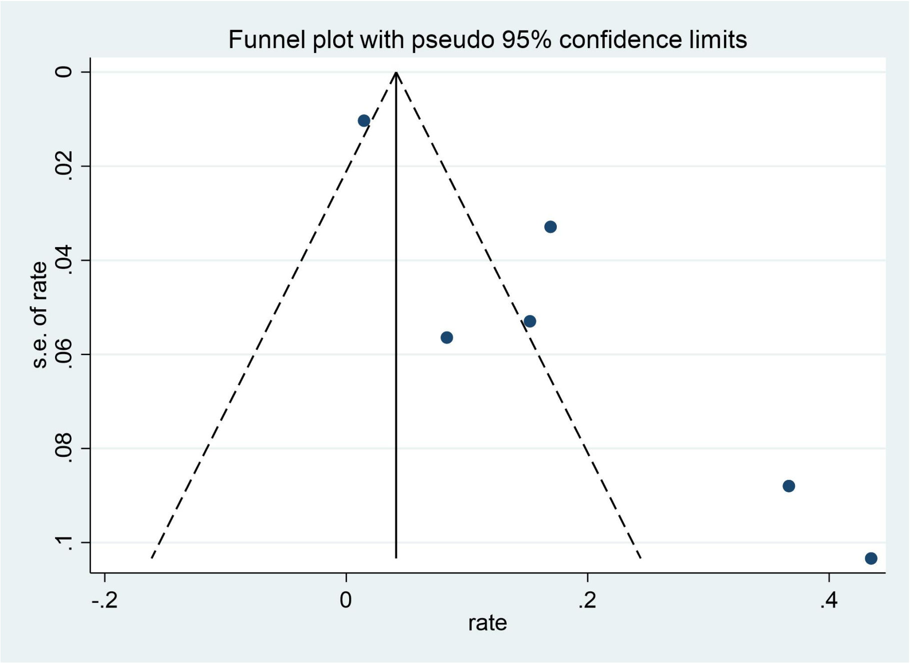

Supplementary Figure 4. Funnel plot showing no publication bias for the studies reporting MPGN.

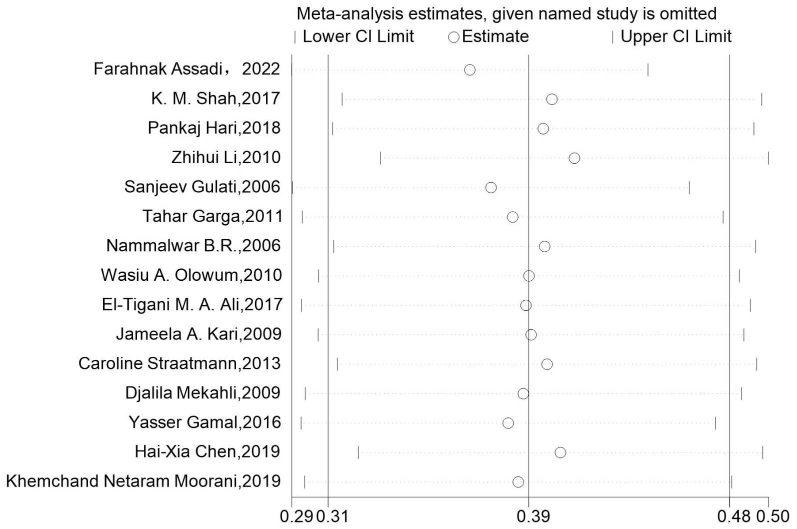

Supplementary Figure 5. Sensitivity analysis in the meta-analysis of FSGS prevalence rates

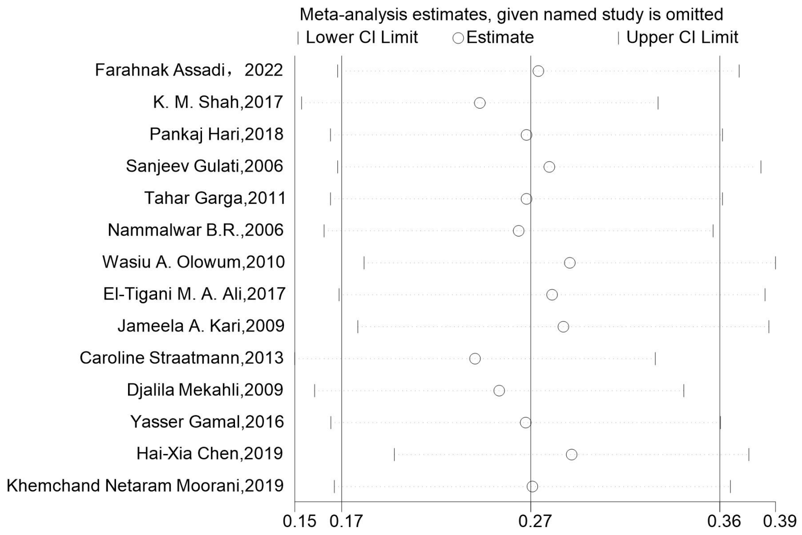

Supplementary Figure 6. Sensitivity analysis in the meta-analysis of MCD prevalence rates

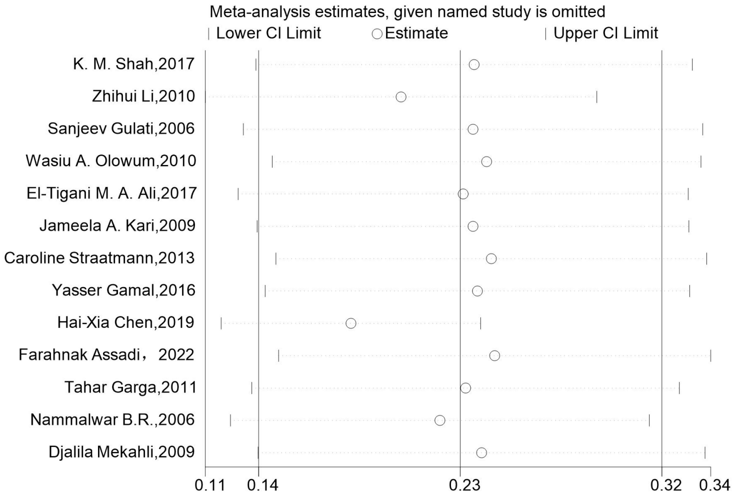

Supplementary Figure 7. Sensitivity analysis in the meta-analysis of MesPGN prevalence rates

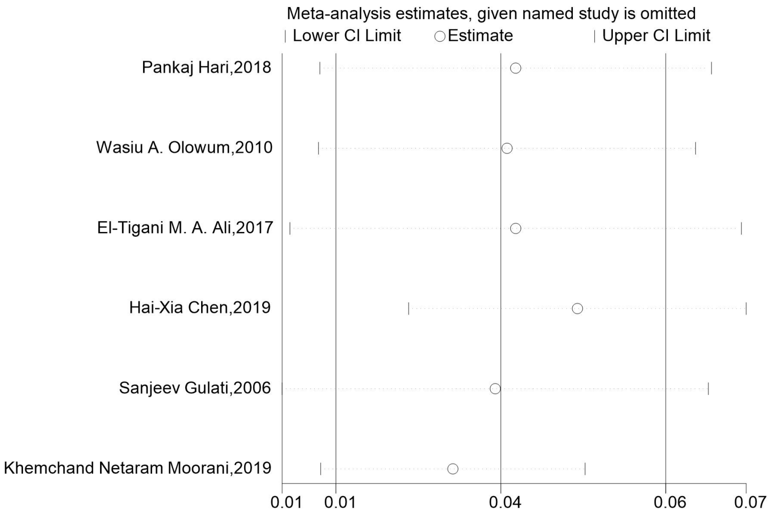

Supplementary Figure 8. Sensitivity analysis in the meta-analysis of MN prevalence rates

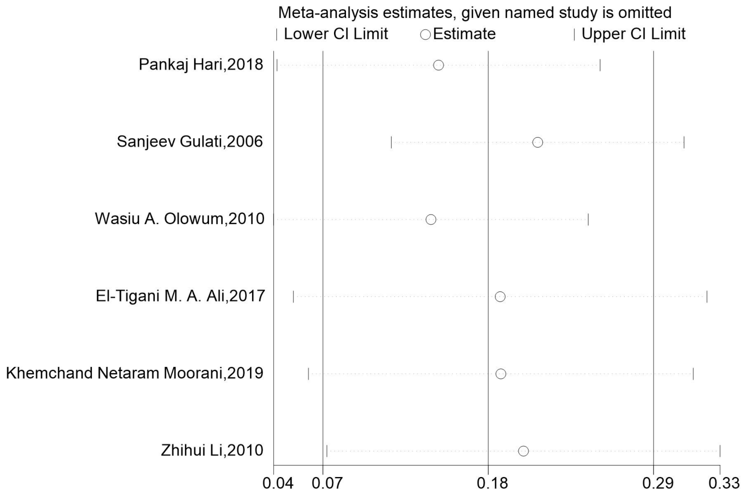

Supplementary Figure 9. Sensitivity analysis in the meta-analysis of MPGN prevalence rates

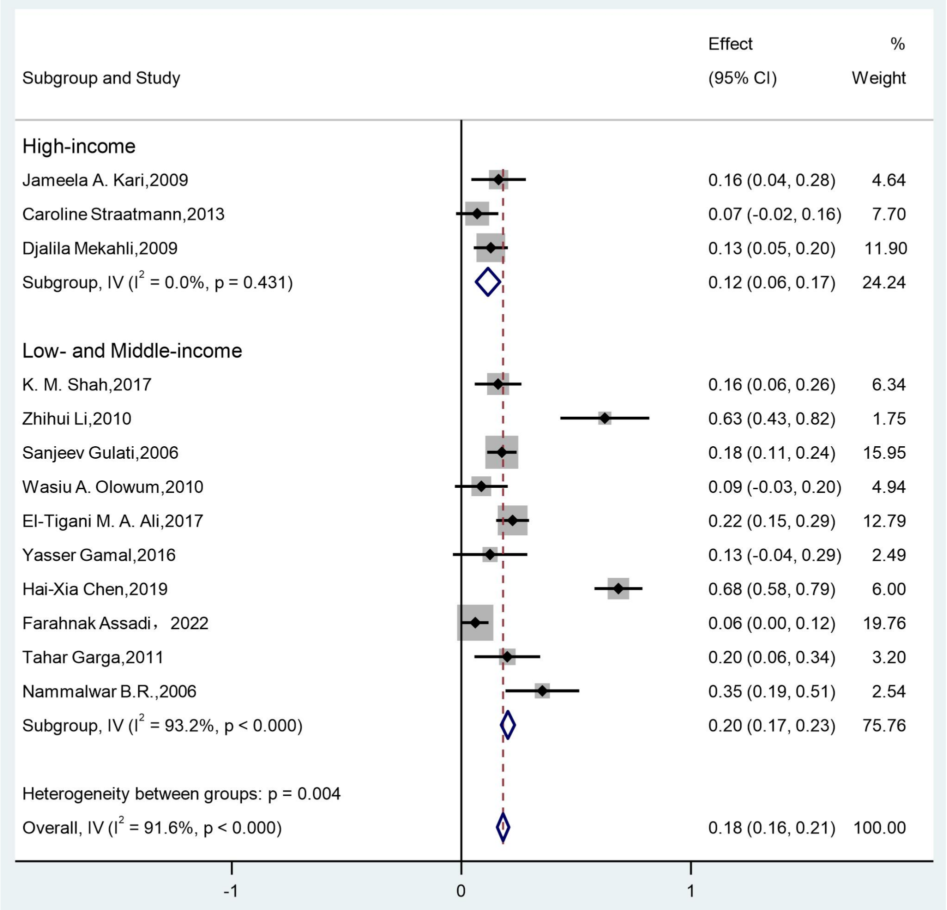

Supplementary Figure 10. Subgroup analysis of prevalence of MesPGN in children by "country income level."

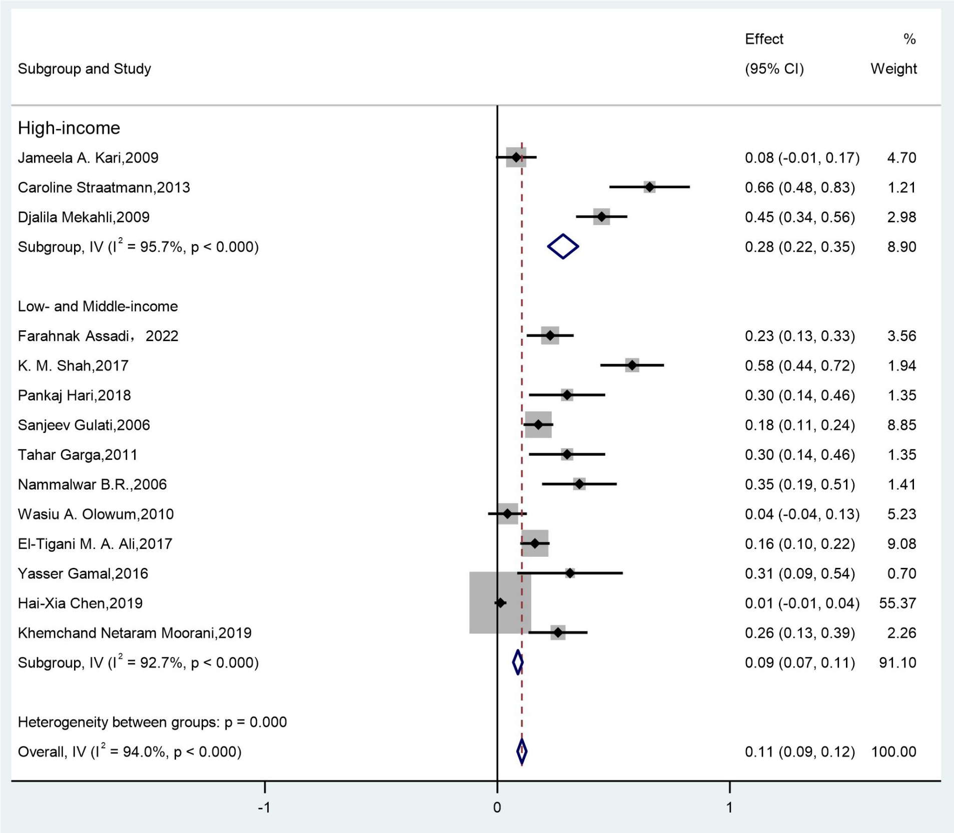

Supplementary Figure 11. Subgroup analysis of prevalence of MCD in children by "country income level."
