# Supplementary material for: Prevalence of histopathological subtypes associated with steroid-resistant nephrotic syndrome in children: a systematic review and meta-analysis
Source: Front Immunol. 2025 Nov 20;16:1647608. doi: 10.3389/fimmu.2025.1647608 (PMC12675457; doi:10.3389/fimmu.2025.1647608)
Supplement: Supplementary file 2 [file Table1.docx]

**Supplementary Table 1.** Quality assessment results for the included studies.

| Study | Checklist | | | | | | | | | Overall |
| --- | --- | --- | --- | --- | --- | --- | --- | --- | --- | --- |
|  | Q1 | Q2 | Q3 | Q4 | Q5 | Q6 | Q7 | Q8 | Q9 |  |
| Farahnak Assadi，2022 | Y | Y | Y | Y | Y | Y | Y | Y | Y | 9 |
| K. M. Shah,2017 | Y | Y | N | Y | Y | Y | Y | Y | N | 7 |
| Pankaj Hari,2018 | Y | Y | N | Y | Y | Y | Y | Y | Y | 8 |
| Zhihui Li,2010 | Y | N | Y | Y | Y | Y | Y | Y | Y | 8 |
| Sanjeev Gulati,2006 | Y | N | Y | Y | Y | Y | Y | Y | Y | 8 |
| Tahar Garga,2011 | Y | N | N | Y | Y | Y | Y | Y | N | 6 |
| Nammalwar B.R.,2006 | Y | N | N | Y | Y | Y | Y | Y | Y | 7 |
| Wasiu A. Olowum,2010 | Y | N | Y | Y | Y | Y | Y | Y | N | 7 |
| El-Tigani M. A. Ali,2017 | Y | N | N | Y | Y | Y | Y | Y | N | 6 |
| Jameela A. Kari,2009 | Y | N | N | Y | Y | Y | Y | Y | N | 6 |
| Caroline Straatmann,2013 | Y | N | N | Y | Y | Y | Y | Y | Y | 7 |
| Djalila Mekahli,2009 | Y | N | N | Y | Y | Y | Y | Y | Y | 7 |
| Yasser Gamal,2016 | Y | N | N | Y | Y | Y | Y | Y | Y | 7 |
| Hai‑Xia Chen,2019 | Y | N | N | Y | Y | Y | Y | Y | Y | 7 |
| Khemchand Netaram Moorani,2019 | Y | N | N | Y | Y | Y | Y | Y | Y | 7 |

**Quality Assessment Questions:**

**Q1:** Was the sample frame appropriate to address the target population?

**Q2:** Were study participants sampled in an appropriate way?

**Q3:** Was the sample size adequate?

**Q4:** Were the study subjects and the setting described in detail?

**Q5:** Was the data analysis conducted with sufficient coverage of the identified sample?

**Q6:** Were valid methods used for the identification of the condition?

**Q7:** Was the condition measured in a standard, reliable way for all participants?

**Q8:** Was there appropriate statistical analysis?

**Q9:** Was the response rate adequate, and if not, was the low response rate managed appropriately?

**Note:**Y = yes;N= not met
